# Mandatory Flu Vaccine for Healthcare Workers: Not Worthwhile

**DOI:** 10.1093/ofid/ofy214

**Published:** 2019-04-17

**Authors:** Michael B Edmond

**Affiliations:** Chief Quality Officer, University of Iowa Hospitals and Clinics

**Keywords:** healthcare workers, influenza vaccine, infection prevention

## Abstract

In 2010, the Society for Healthcare Epidemiology published a recommendation that annual influenza vaccination of healthcare workers be made a condition of employment despite no high-level evidence to support this recommendation. A better strategy for reducing the transmission of respiratory viruses in the healthcare setting would be to encourage vaccination and reduce presenteeism, which is very common among healthcare workers with influenza-like illness. In a hospital with a baseline vaccination compliance of 70%, reducing presenteeism by 2% has the equivalent impact of mandating vaccination in terms of the number of healthcare workers with influenza-like illness at work. Expectations for compliance with interventions to improve the quality of care should be correlated tightly to the underlying evidence to support the intervention, reserving mandates for interventions with very high quality supporting evidence.

## Teaser

Mandatory influenza vaccination of healthcare workers is not supported by evidence. Efforts at reducing presenteeism are likely to be more effective than mandatory vaccination.

Annual vaccination of healthcare workers against influenza has been recommended by the Centers for Disease Control and Prevention since 1984 [1]. In 2005, the Society for Healthcare Epidemiology (SHEA) published a position paper that stated that “all healthcare workers should receive influenza vaccine annually unless they have a contraindication to the vaccine or actively decline vaccination.” [2] Five years later, SHEA issued a revised position paper, which recommended that annual influenza vaccination be made a condition of employment for healthcare workers [3]. Other professional societies subsequently endorsed SHEA’s “get vaccinated or get fired” recommendation. This paper will explore the underlying supporting evidence (or lack thereof) for mandatory vaccination, its potential impact on infection of healthcare workers and patients, and whether an alternative approach, reducing presenteeism, may be more effective than mandatory vaccination.

In this paper, I will make 3 key arguments: (1) influenza vaccine is a weakly effective but safe vaccine; (2) high-level evidence demonstrating influenza reduction in hospitalized patients by vaccinating healthcare workers does not exist; and (3) threatening a person’s employment for noncompliance with this intervention is not warranted based on the available evidence.

Influenza-like illness (ILI) is caused by over 200 viruses and bacteria. Only 10% of cases of ILI are due to influenza viruses. However, influenza and non-ILI may be clinically indistinguishable. The primary goal of any infection prevention program to prevent transmission of ILI due to any pathogen from healthcare workers to patients and coworkers should be to reduce the number of healthcare workers who are present at work while infected to the lowest extent possible. This can be achieved via prevention of influenza infection in healthcare workers (through vaccination and postexposure prophylaxis) and for all ILI by limiting exposure to infected persons by preventing infected healthcare workers from working.

## PRESENTEEISM

Presenteeism is defined as working while ill. This is a problem across many industries, but it has particular impact in healthcare because infected workers may transmit infection to patients who have multiple comorbidities, are immunosuppressed, and/or are at risk of severe complications. Two recent studies demonstrate the high prevalence of presenteeism. In a national study of approximately 2000 healthcare workers, 41% of those experiencing ILI reported working while ill [4]. Presenteeism was 63% for physicians and 47% for nurses. Another study conducted in a major tertiary care medical center, which surveyed healthcare workers caring for hospitalized internal medicine and transplant patients, found that presenteeism was 92% in those with ILI [5]. Despite high rates of presenteeism, hospital infection prevention programs put little emphasis on keeping ill employees from work, instead basing their influenza prevention efforts primarily on attempting to achieve high rates of compliance with vaccination.

One driver of presenteeism is paid time off. This is an increasingly common human resource practice in which a bank of combined leave time is used for personal days, sick days, and vacation. For some employees, a perverse incentive exists to work while ill to avoid using time off so that vacation time can be maximized [6]. More importantly, particularly for physicians, are strong professionalism forces (ie, feelings of obligations to patients and colleagues) that compel attendance when ill. In a survey of physicians and advanced practice clinicians at a large academic children’s hospital, 83% reported working while ill in the past year, despite 95% reporting that this put patients at risk [7]. Over 90% of respondents worked while ill because they did not want to let their colleagues or their patients down.

## INFLUENZA VACCINE

The influenza vaccine has been approved and available since the mid-1940s. Approximately 60% of adults in the United States receive the vaccine yearly [8]. It is very safe. The only significant adverse effects are allergic reactions, primarily in persons allergic to eggs, and a possible association with Guillain-Barré Syndrome, occurring at a rate of 1–2 additional cases per million vaccinations [9].

**Figure 1.**
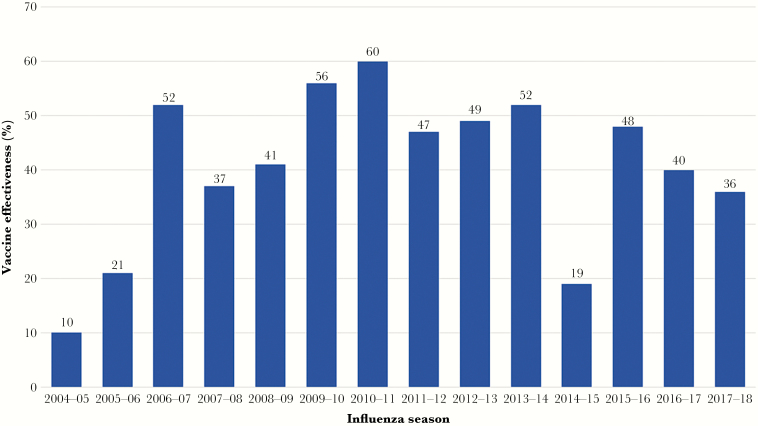
Effectiveness of the influenza vaccine by influenza season.

The influenza vaccine is modestly effective. The mean effectiveness over the most recent 14 consecutive influenza seasons beginning in 2004–2005 was 41% (range, 10%–60%; Figure 1) [10]. This stands in sharp contrast to effectiveness rates for other commonly used vaccines in clinical practice, many of which exceed 90%. A large meta-analysis of influenza vaccine effectiveness evaluated placebo (or no intervention) controlled randomized trials and quasi-randomized trials in healthy adults (ages 16 to 65 years old). This analysis included 52 studies with 80 000 subjects. The relative risk reduction for influenza infection associated with influenza vaccine was 59%. However, the absolute risk reduction was only 1.4% (2.3% infected among unvaccinated persons versus 0.9% infected among vaccinated). Moreover, this study also found no significant effect of vaccination on working days lost or hospitalization [11].

In the healthcare setting, the direct effect of vaccination is reduction of influenza rates in healthcare workers. The indirect effect is the reduction of influenza rates in patients due to vaccinating healthcare workers. Two meta-analyses evaluating the impact of influenza vaccination of healthcare workers on outcomes in patients have been published. Both studies evaluated the same 4 cluster randomized trials that were performed in long-term care facilities. Of note, these same 4 studies were used by SHEA to recommend mandating influenza vaccination of healthcare workers. Ahmed et al [12] found a significant reduction in all-cause mortality (−44 per 1000 patients) in facilities where healthcare workers were offered influenza vaccination. The quality of evidence was graded as moderate. There was also a significant reduction in ILI among patients (−68 per 1000 patients) using evidence that was graded as low. There was no significant difference in all-cause hospitalization or laboratory-confirmed influenza [12]. The meta-analysis by Thomas et al [13] showed no significant difference in influenza, lower respiratory tract infection, or hospitalization for respiratory illness. Influenza-related mortality and all-cause mortality were not assessed because the data were not pooled due to inconsistencies in the size and direction of the risk differences [13]. Thus, there is no evidence to date that vaccinating healthcare workers will indirectly reduce influenza infection in patients in long-term care settings.

It is also important to point out that there are no randomized controlled trials evaluating influenza vaccination of healthcare workers in acute care hospitals, with the exception of a Dutch study in which 6 hospitals were randomized [14]. Three had an intervention to increase vaccination rates in healthcare workers and 3 did not. Significantly higher vaccination rates were demonstrated in the intervention hospitals, although even in the intervention hospitals vaccination compliance was quite low (<33%). The patient outcomes were divided into adult and pediatric patients, and the outcomes reported for patients were influenza and/or pneumonia and pneumonia. Influenza was not a reported outcome. Thus, the influenza rates cannot be determined. For adults there was a 50% reduction in influenza and/or pneumonia in intervention hospitals. For children, there was no difference between the intervention and control hospitals. It is worth noting that the intervention hospitals had significantly higher healthcare worker absenteeism rates.

**Figure 2.**
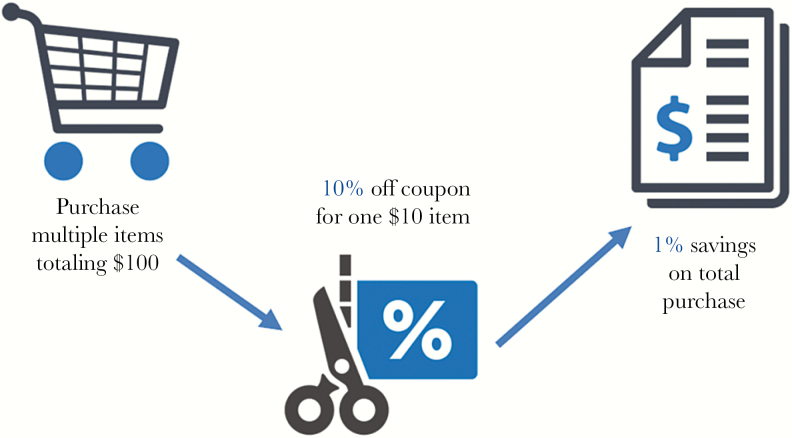
A simple example of the principle of dilution.

De Serres et al [15] recently critically analyzed the 4 cluster randomized trials that formed the basis of the SHEA recommendation. They found multiple sources of bias (eg, mortality was accrued and attributed before the onset of influenza in the community), wildly inaccurate estimates of numbers needed to vaccinate (by as much as 4000-fold), and all 4 studies violated the principle of dilution. This basic mathematical principle holds that a percentage reduction for a specific outcome will always be greater than for a less specific outcome attributed to an intervention. That is, the percentage reduction in laboratory-confirmed influenza due to vaccination should be greater than the attributed percentage reduction in ILI (because influenza accounts for only a small fraction of ILI cases), which should be greater than the attributed percentage reduction in all-cause mortality. This can easily be explained with the example of a grocery store coupon (Figure 2). Suppose you have 1 single-use 10%-off coupon for a $10 item and your entire purchase, which includes several other items, totals $100. You will save $1 in toto (10% off the coupon discounted item, which translates to 1% off your total purchase). In this scenario, it would be impossible for the discount on your entire purchase to be >10%. However, in the 4 cluster randomized trials, moving from more specific outcomes (laboratory-confirmed influenza) to less specific (death), the percentage reductions attributed to influenza vaccination of healthcare workers were greater, which cannot be possible.

## ANALYSIS OF INFLUENZA-LIKE ILLNESS CONTROL STRATEGIES

To demonstrate the impact of mandatory vaccination and an alternative strategy of reducing presenteeism in healthcare workers, we will use a simple model of multiplicative probability to determine the number of healthcare workers who are present at work with ILI. Influenza-like illness is used as the outcome because the pathogens responsible for this syndrome are also transmissible to patients. The assumptions for the model include the following: there is a workforce of 5000 healthcare workers in a hypothetical hospital. The attack rate of influenza in a nonpandemic season is 7% [16], and the attack rate for noninfluenza ILI is 16% [17]. The hospital has a baseline voluntary influenza vaccination rate of 70% [18] and a baseline presenteeism rate for ILI of 41% [4]. The effectiveness of influenza vaccine is 41% [10]. The vaccination rate after issuing a mandate for vaccinations rises to 98%. Asymptomatic and presymptomatic transmission of influenza from healthcare workers to patients is negligible [19]. With these assumptions, we can now calculate the number of healthcare workers at work with ILI under various scenarios (Table 1).

**Table 1.** Impact of Strategies to Reduce Influenza-Like Illness (ILI) in Healthcare Workers

		Baseline	Alternative Strategies To Reduce ILI		
				Voluntary Vaccination + Reduce Presenteeism^a^	
Parameters		Voluntary Vaccination	Mandatory Vaccination	2%	10%
Vaccination rate		70%	98%	70%	70%
Presenteeism rate		41%	41%	39%	31%
Total healthcare workers		5000	5000	5000	5000
• Vaccinated workers		3500	4900	3500	3500
• Unvaccinated workers		1500	100	1500	1500
Workers with illness	Non-flu ILI	800	800	800	800
	Unvaccinated with flu	105	7	105	105
	Vaccinated with flu	145	202	145	145
	Total with ILI	1050	1009	1050	1050
	At work with ILI	430	414	409	325

^a^Presenteeism percentage reductions shown are absolute reductions.

In the baseline scenario, with a vaccination rate of 70% and a presenteeism rate of 41%, the number of healthcare workers predicted to be at work with ILI is 430. In the scenario where vaccination is mandated and the vaccination rate rises to 98% while the presenteeism rate remains the same at 41%, 414 healthcare workers are predicted to come to work with ILI. In the third scenario, the vaccination rate remains at 70%, but the presenteeism rate is reduced by 2 percentage points to 39%. Now we see that the number of healthcare workers at work with ILI is expected to be 409. Thus, a small reduction in presenteeism produces an outcome of equivalent impact to mandating vaccination. If presenteeism were reduced by 10 percentage points to 31%, the number of ill workers in the hospital would be significantly reduced to 325. In our hypothetical workforce of 5000, for every 10% improvement in vaccination compliance, 6 fewer healthcare workers will be present at work with ILI, demonstrating the minor impact of vaccination due to the vaccine’s relatively low effectiveness and influenza accounting for a minority of ILI cases. However, for every 10% reduction in presenteeism, 105 fewer healthcare workers will be present at work with ILI. It is important to note that this model does not take into account herd immunity derived from vaccination of healthcare workers and hospitalized patients, which would reduce the impact of mandatory vaccination.

Influenza vaccination of healthcare workers is a vertical infection prevention strategy that is unipotent and pathogen based [20]. In contrast, reducing presenteeism is a horizontal strategy that is multipotent, cutting across many pathogens that may be transmitted from healthcare workers to patients, and is population based. The philosophical underpinning of vertical strategies is exceptionalism, a belief that the targeted pathogen is more important than other pathogens transmitted via the same mechanism. In contrast, horizontal strategies are utilitarian with a goal to reduce all infections, which is more in line with the values of patients.

Some hospitals have required unvaccinated healthcare workers to wear masks during the influenza season. For influenza, this is not an evidence-based practice. If one were to assume that masking asymptomatic workers is effective, given the poor performance of the vaccine, universal masking would make more sense. In addition, as the proportion of vaccinated workers increases, the proportion of infected vaccinated workers increases disproportionately. As shown in Table 1, even with a vaccination rate of 70%, approximately 60% of workers with influenza will have received the vaccine. With universal vaccination, 97% of influenza cases will occur in vaccinated workers who will not be masked. Thus, masking unvaccinated workers is most likely punitive and coercive rather than a well reasoned strategy for reducing transmission in the healthcare setting.

For any quality-improvement intervention, expectations for compliance must be tightly correlated to the strength of the evidence. For influenza vaccination, there is biologic plausibility that supports strongly recommending and encouraging healthcare workers to get vaccinated, but clearly there is not enough evidence to mandate vaccination and threaten employment. As De Serres et al [15] note, “an intuitive sense that there may be some evidence in support of patient benefit is insufficient scientific basis to ethically override individual healthcare worker rights.” If hospitals were truly serious about protecting their patients, they would develop programs to reduce presenteeism in conjunction with a voluntary vaccination program, which would have a much greater impact on infection transmission in the hospital.

## CONCLUSIONS

Just as we must defend vaccines from false claims of adverse effects, we must also truthfully acknowledge their limitations and shape our policy on science not opinion. Mandates must be reserved for interventions supported by the highest quality evidence. In the long run, use of soft power rather than a dogmatic approach without high-level supporting evidence is more likely to result in a workforce with a greater likelihood of compliance with other behaviorally based infection prevention interventions. The SHEA’s recommendation for making influenza vaccination a condition of employment has no more evidence to support it today than when it was published in 2010, and it should be withdrawn.
